# Decision processes in 3D structural MRI schizophrenia classification evaluated with saliency maps

**DOI:** 10.1038/s41598-026-57667-z

**Published:** 2026-06-13

**Authors:** Julia Jelitzki, Alexandra Reichenbach, Alexander Windberger

**Affiliations:** 1 Center for Machine Learning, Heilbronn University, Heilbronn, Germany; 2 Medical Faculty Heidelberg, University of Heidelberg, Heidelberg, Germany; 3 Faculty of Informatics, Heilbronn University, Heilbronn, Germany

**Keywords:** Deep learning, Schizophrenia, Structural magnetic resonance imaging, Explainable AI, Grad-CAM, Saliency map, Diagnostic markers, Schizophrenia, Diagnostic markers, Image processing, Machine learning, Schizophrenia

## Abstract

Clinical decision support systems for psychiatric disorders such as schizophrenia can benefit from machine learning models based on neuroimaging data for objective diagnosis, prognosis, and effective treatment selection. Deep learning (DL) models promise to be suitable for this task since they can detect complex patterns in images without the need for prior information about candidate regions. Their downside, however, is the lack of transparency about the decision process. Explainable AI methods address this problem and might be helpful in the clinical translation of DL applications as well as potential biomarker indication. The current study qualitatively and quantitatively evaluates seven DL architectures frequently employed in medical image analyses with gradient-weighted class activation mapping (Grad-CAM) for plausibility and finds that only two of the seven models base their decisions in a schizophrenia classification task on plausible structural brain information, despite similar classification performance. Furthermore, we develop an approach to translate the saliency maps from the Grad-CAM into universally interpretable anatomical markers of schizophrenia and find candidate regions corresponding to known markers of schizophrenia. To conclude, this study demonstrates the necessity of using explainable methods alongside DL approaches and the feasibility to derive biomarkers with such methods.

**Supplementary Information:**

The online version contains supplementary material available at 10.1038/s41598-026-57667-z.

## Introduction

Psychiatric disorders such as schizophrenia, depression, or anxiety disorders are characterized by high heterogeneity in symptoms and wide-spread structural as well as functional alterations of the brain^1,2^. Neuroimaging, especially (functional) magnetic resonance imaging ((f)MRI) provides information about those alterations and fosters insight into the pathologies of the disorders. Quantification of those alterations might also be used as biomarkers in a clinical setting^3^. Advances in application of machine learning (ML) techniques on neuroimaging data show promise in clinical decision support for diagnosis or prognosis, therapy decision, and treatment development^4,5^. Classical ML-approaches for these tasks usually process extracted features based on biomarkers or other expert knowledge^6,7^. Deep learning (DL) models circumvent this a priori selection by incorporating feature selection mechanisms operating on the data either in a rich, high-dimensional feature space or in its original form^8–10^. Current improvements in the field of deep learning e.g., specialized convolutional neural networks (CNN) architectures for medical images, enable effective detection of complex structural alterations^8,11–13^. However, those methods come with their own impediments. The complexity of deep learning architectures makes them data greedy, requiring large data sets for training. Medical image data sets are several magnitudes smaller than the image data sets that brought deep-learning based image analysis their breakthrough^14^. Counter-intuitively, small data sets can yield very good performance, which is frequently a sign of overfitting to the specific data set^12,15^. This problem can be addressed with transfer learning, i.e., using a larger, less specific data set or employing weights from adjunct fields of application for training and only re-adjusting some of the downstream layers^16^.

Still, classical DL models lack innate ability to explain their decision processes or require a specific, ante-hoc model design to capture human-understandable information and high quality data patterns alike. In order to illuminate the behavior of pre-existing models, post-hoc explainable artificial intelligence (XAI) methods allow the clarification of decisions in DL-based clinical decision support systems. Although those methods are known to strengthen the trust of all stakeholders in such systems, post-hoc XAI methods are occasionally used but not yet standard practice in medical DL research^17,18^. If deployed, one of the most commonly used techniques is the image-based Gradient-Weighted Class Activation Mapping (Grad-CAM)^19^. This local method generates saliency maps for individual test images with respect to a specific output class. Two recent studies used Grad-CAM saliency maps to evaluate the plausibility of their schizophrenia classifiers^20,21^. The advantage of Grad-CAMs is their explanation on the individual level which provides transparent and intuitive information in a diagnostic or prognostic clinical setting. These local explanations, however, cannot provide reliable information on reoccurring, consistent patterns in the dataset, let alone in the disease as a whole. To derive this kind of generalized explanation, information across multiple patient maps has to be aggregated.

This work explores the necessity of transparent clinical AI decision support and evaluates the practicality of XAI methods, more specifically saliency maps derived with Grad-CAMs, for providing this transparency. Furthermore, we offer an approach to derive neuroanatomical biomarker candidates of a psychiatric disorder across patient saliency maps (Fig. 1). In the first stage (classification), we train and evaluate seven DL-architectures frequently used in the field of medical image processing to separate 3D MR images of schizophrenia patients from healthy controls. Sequence 1 (Seq1, inspired by VGG16^22^ and OhNet (OhNet)^23^, were trained from scratch. Med3D^24^, BrainID^25^, RiekeNet^26^, Mixed Convolution Network (Mixed Conv)^27^, and ResNet18^27^ are publicly available architectures pre-trained on diverse training sets. We hypothesize that classifiers extracting physiological correlates of the disorder should localize anatomically plausible features from the images and eventually converge across architectures. In the second stage (local explanation), we evaluate the performance of the architectures with regard to the classification task, and their plausibility based on quantitative metrics derived from Grad-CAM saliency maps. The most suitable classifiers based on these evaluations are then selected for further investigations. In the third stage (global explanation), we first derive locations that differ robustly between clinical groups based on statistical comparisons within classification architectures. Secondly, we intersect those locations to derive robust regions across classifiers. Mapping the locations derived from the individual classifiers as well as their intersections on the corresponding brain areas provides candidate regions for schizophrenia pathology and potential anatomical biomarkers. This approach constitutes a general method that allows the transition between local saliency map explanations and a global statistical evaluation indicating brain areas relevant to psychiatric biomarkers.

## Results

**Fig. 1:**
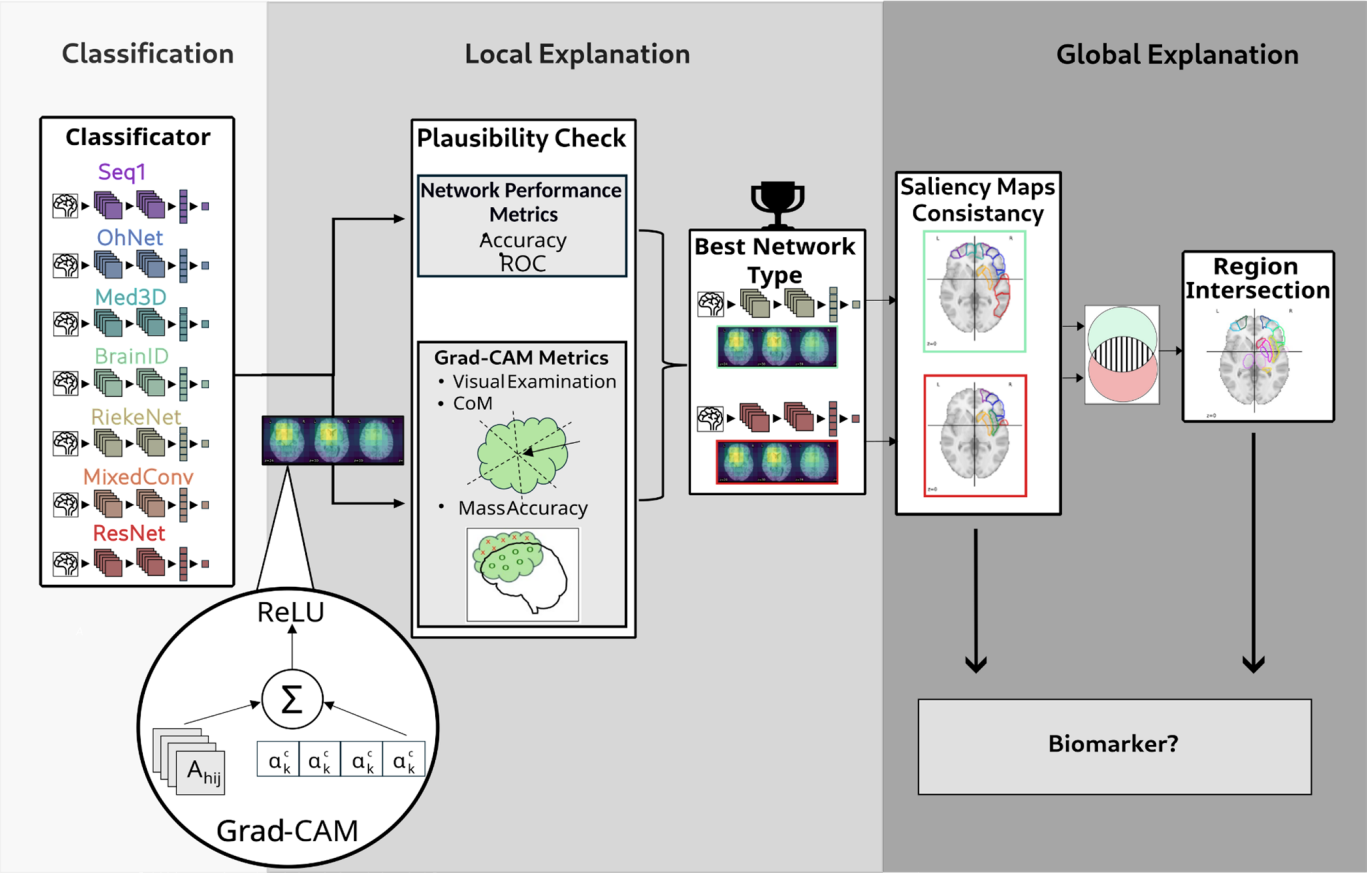
Three-stage process to explain the decision process in diagnosis classifiers with Grad-CAM and derive neuroanatomical underpinnings of the disorder. (1) Classification: training of seven different DL architecture types: Sequence 1 (Seq1, inspired by VGG16^22^ and OhNet (OhNet)^23^, were trained from scratch. Med3D^24^, BrainID^25^, RiekeNet^26^, Mixed Convolution Network (Mixed Conv)^27^, and ResNet18^27^ are publicly available architectures pre-trained on diverse training sets ranging from human motion clips (Mixed Conv, ResNet) over mixed or synthesized medical imaging modalities (Med3D, BrainID) to sMRI data for Alzheimer’s classification (RiekeNet). (2) Local Explanations: Plausibility check for all architecture types with evaluation of classification performance and three subject-specific Grad-CAM metrics (visual saliency map inspection, center of mass (CoM) deviation analysis, and mass accuracy as an estimation of Grad-CAM accuracy). (3) Global Explanation: Derivation of robust differences between patient and control class saliency maps for the most promising two network types. Detection of stable regions across subject Grad-CAMs within each architecture and across all architectures. *sMRI* structural magnetic resonance imaging; *ReLU* rectified linear unit; Grad-CAM: gradient-weighted class activation mapping; *ROC* receiver-operating characteristic.

### Classification performance

**Fig. 2:**
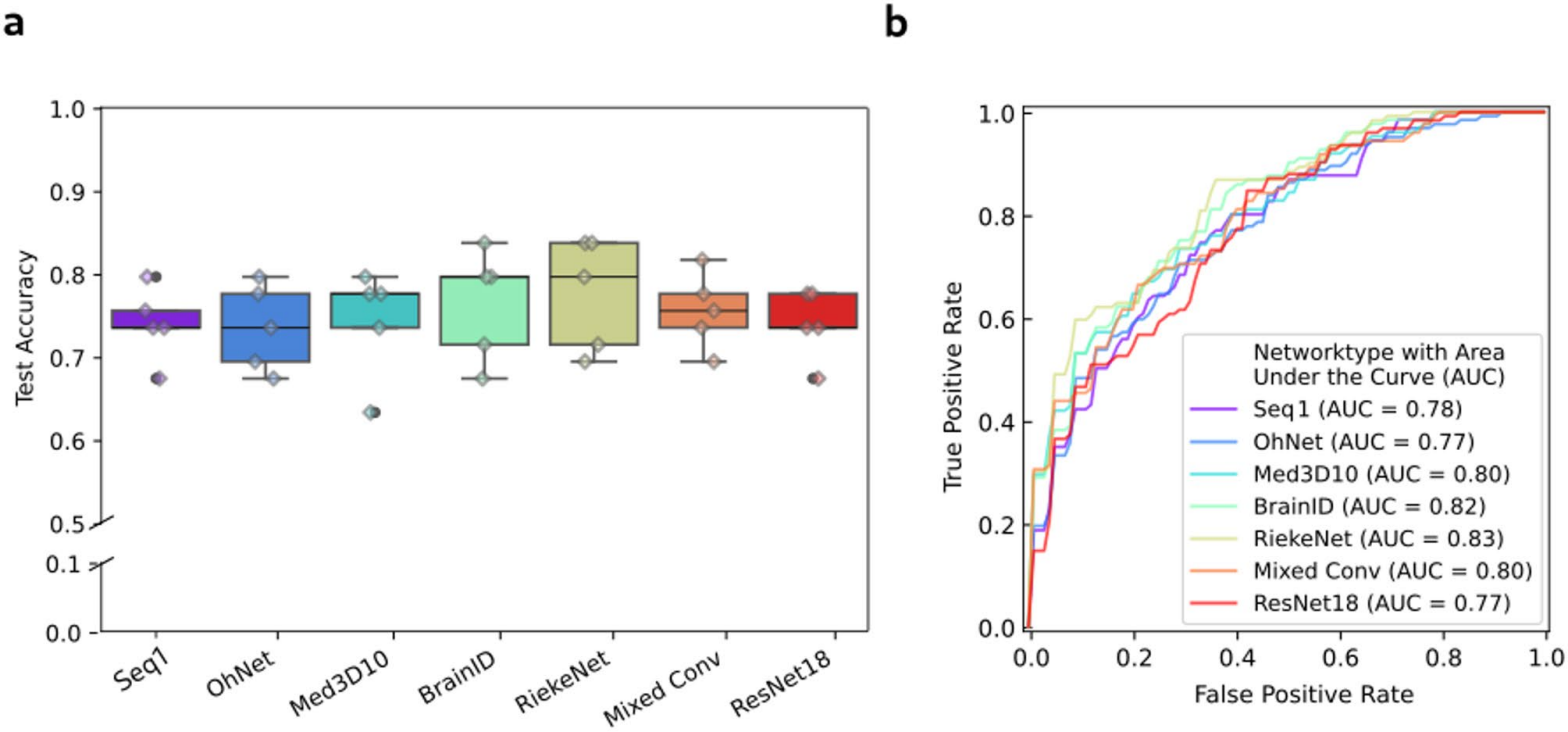
Classification performance for all architecture types. (**a**) highest network accuracy per stratification run and (**b**) average receiver operating characteristic (ROC) curve with associated area under the curve (AUC), both collected in a 5-fold stratification process.

In the first stage (Fig. 1, classification), we train seven commonly used CNN architectures for MRI image classification on structural T1-weigthed MR images of 192 adult brains (101 schizophrenia patients and 91 healthy control subjects) from the MCIC collection^28^. The models were either trained from scratch (Seq1, OhNet) or with transfer learning (Med3D10, BrainID, RiekeNet, MixedConv, ResNet18) with 5-fold cross-validation. On average, all classifiers exhibited a stratified classification accuracy well above 70% (Fig. 2a) and area-under-the-curve (AUC) scores of more than 0.75 (Fig. 2b) without significant differences in accuracy (*F*_*6,28*_=0.371, *p*=0.891). These classification accuracies are well within the range of what is expected from an ML-classifier based on structural MRI data^8^.

## Local explanation: Individual saliency map evaluation

**Fig. 3:**
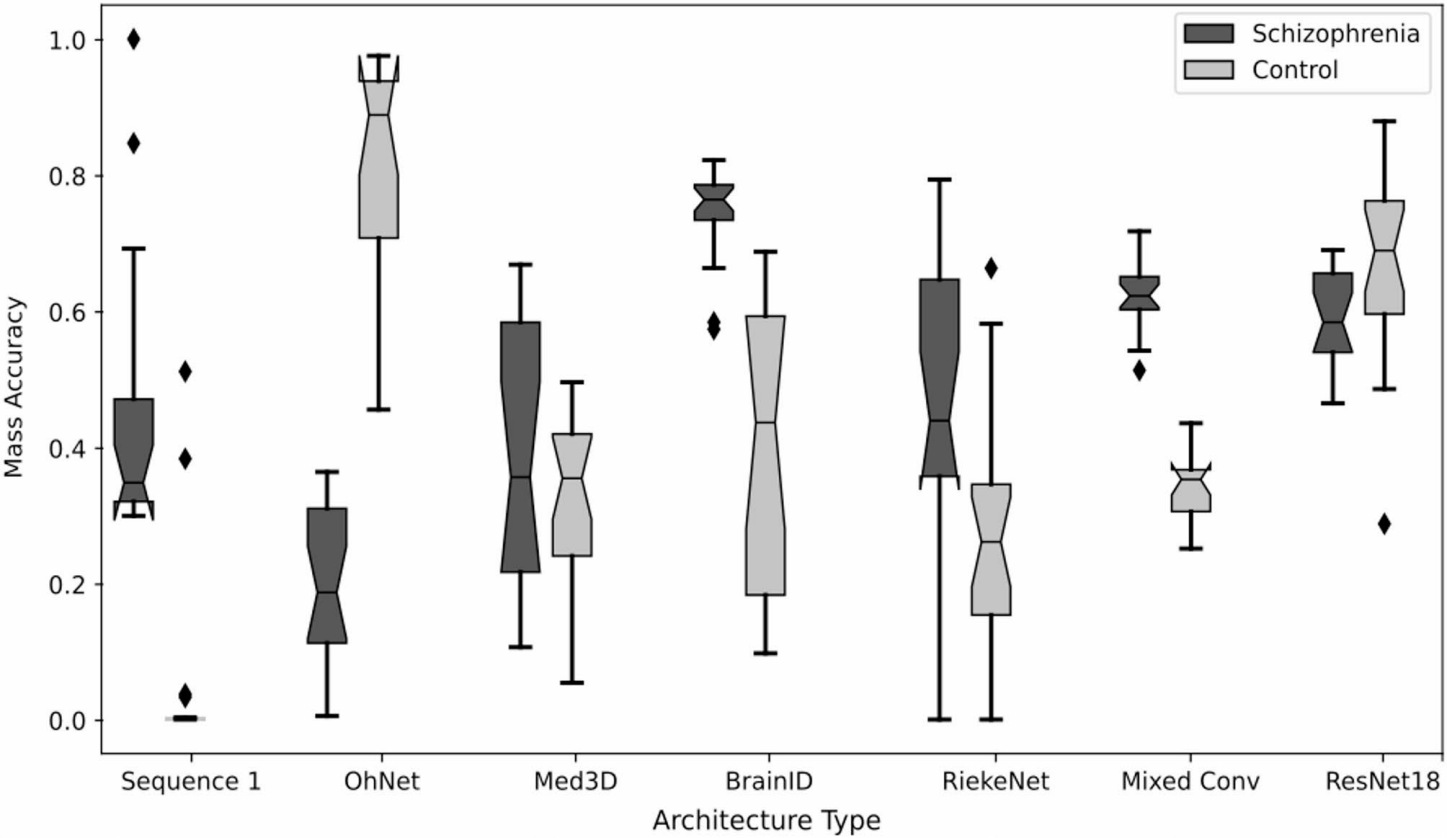
Mass accuracy for all architectures, separated for the two clinical groups. Per architecture type, the Grad-CAM generation was conducted with the best performing example network and was based on at least 17 correctly classified, unseen test set images in every group. Data points outside the median ± 1.5*IQR are depicted as outliers.

In order to make the decision process in the classifiers visible and quantifiable, we generate saliency maps with Grad-CAM for the individual test images. The intensity of the voxel in the saliency map scales with its contribution to the model’s decision. Two metrics are generated to quantify the plausibility of the classifier’s decision process. Mass accuracy (MA) is a ratio representing the accumulated intensity of saliency within the brain vs. outside the brain area^18^ (Fig. 3). We use the whole brain as an area of interest and do not constrain the location to known areas affected in schizophrenia in order to avoid a bias towards current literature. A high portion of attention outside of the brain area points towards overfitting or inconsistency of the learned feature representation. The center of mass (CoM) describes the intensity-weighted average of the voxel positions of a saliency map. In order to describe the groups center’s position in image space, the average vector length and its standard deviation (STD) over the test set is considered (Table 1). Uniformly distributed saliency would yield average CoMs close to the image center. Furthermore, a low standard deviation of CoM across subjects saliency maps indicates convergence across persons in each clinical group and, hence, is preferred over broadly scattered saliency. The former is used in conjunction with qualitative assessment of the averaged saliency maps per CNN architecture since, e.g., two areas localized in point symmetry would also yield a central CoM.

**Table 1:** Average center of mass (µ, relative to image center) and its standard deviation (σ) within the two clinical groups schizophrenia (SZ) and control (C) for all architectures. Two-sided *t*-tests compare vector lengths of CoMs to corresponding image midpoint between schizophrenia and control groups (bold: sign. on *p* < 0.05).

Architecture	SZ µ	SZ σ	C µ	C σ	*p*-value	t-value	df
**Sequence 1**	**6.77**	**2.94**	**28.43**	**14.40**	**1.936e-6**	**−5.98**	**37**
OhNet	6.95	3.38	7.21	3.94	8.339e-1	−0.21	36
**Med3D10**	**17.55**	**4.20**	**12.01**	**9.09**	**2.942e-2**	**2.27**	**37**
**BrainID**	**7.69**	**1.79**	**15.95**	**3.31**	**2.389e-12**	**−10.02**	**39**
RiekeNet	29.00	22.01	24.06	30.03	5.963e-1	0.54	39
Mixed Convolution	1.69	0.59	2.08	0.64	6.078e-2	−1.94	36
**ResNet**	**6.09**	**2.30**	**13.03**	**4.55**	**2.314e-6**	**−5.64**	**35**

**Fig. 4:**
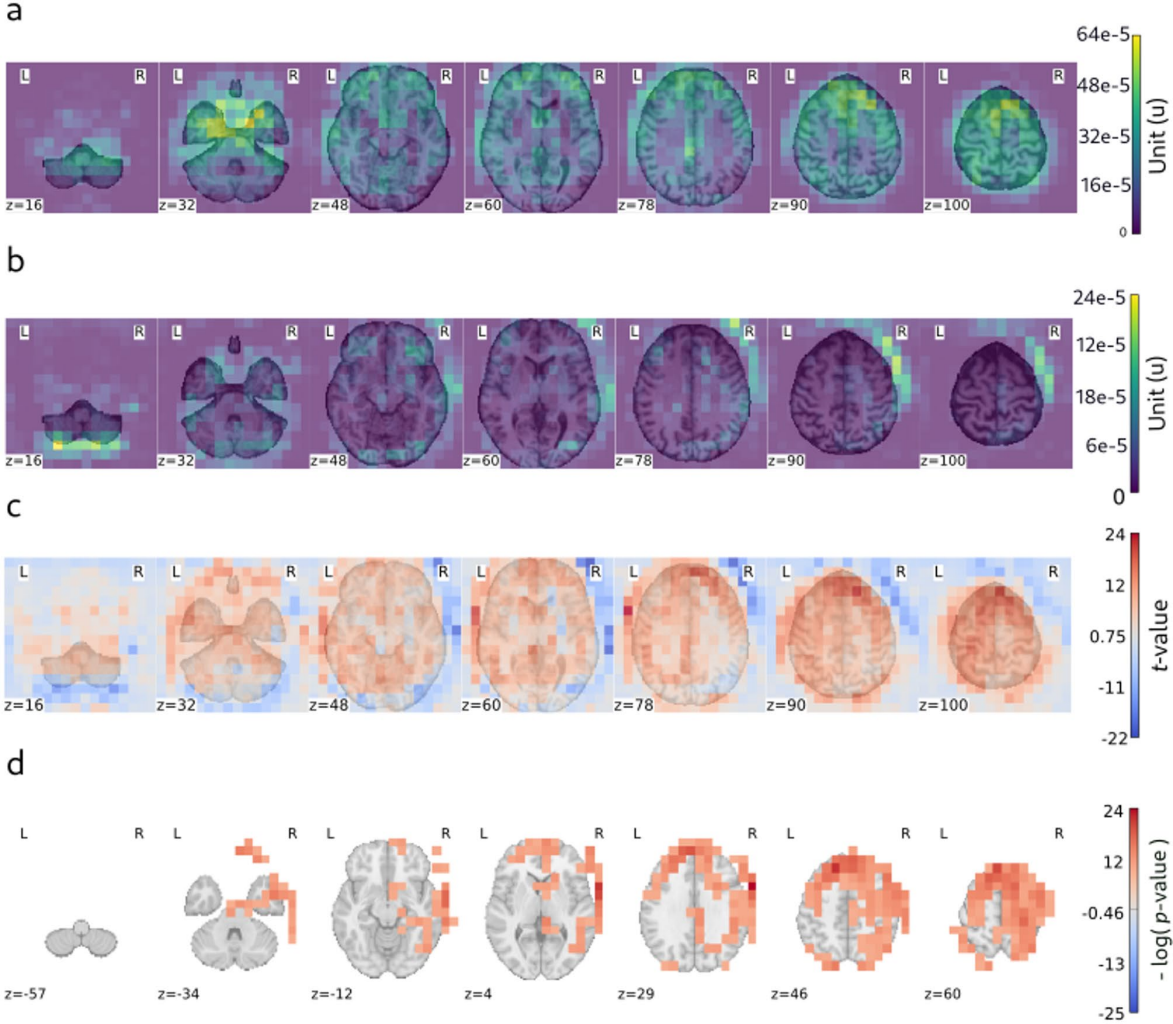
Saliency maps and statistical derivations of the BrainID classifier, overlaid on an exemplary patient image. Average network attention maps in the **(a)** patient and **(b)** control groups. **(c)** Uncorrected *t*-maps (two-sided) contrasting schizophrenia and patient groups. **(d)** Negative log *p*-value maps after threshold-free cluster enhancement (TFCE)^29^* p*-value correction and additional Bonferroni correction on the remaining voxels. The one contiguous cluster comprises 100,743 voxels. All maps were derived with image input of the size 128 × 128 × 128 voxel and thereby result in saliency depictions of the same size. Note that all maps are rather coarse due to the convolutions in the classification networks.

**Fig. 5:**
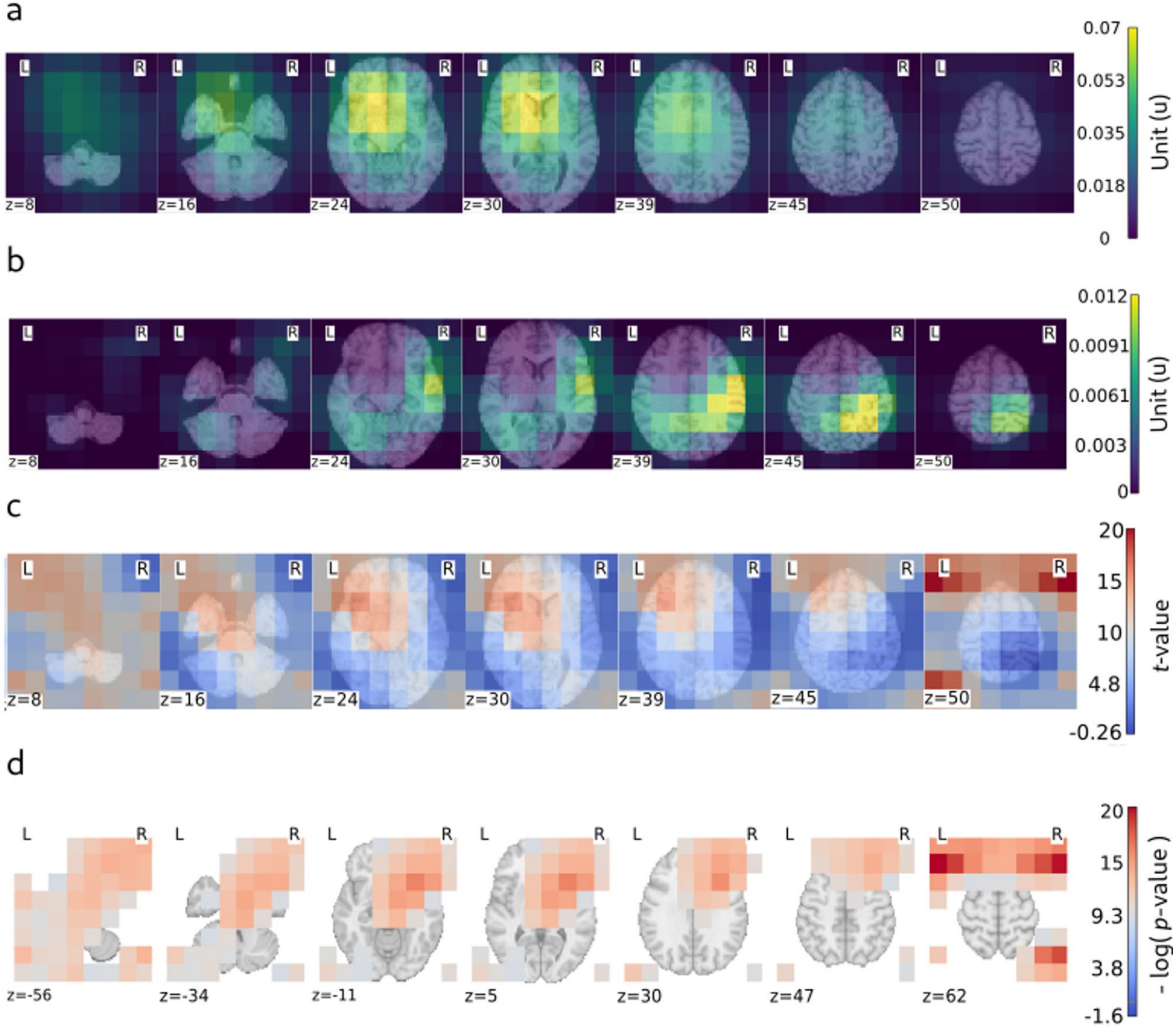
Saliency maps and statistical derivations of the ResNet18 classifier, overlaid on an exemplary patient image. Average network attention maps in the (**a**) patient and (**b**) control groups. (**c**) Uncorrected *t*-maps (two-sided) contrasting schizophrenia and patient groups. (**d**) Negative log *p*-value maps after TFCE *p*-value correction and additional Bonferroni correction on the remaining voxels. The one contiguous cluster comprises 342,354 voxels. All maps were derived with image input of the size 64 × 64 × 64 voxel and thereby result in saliency depictions of the same size. Note that all maps are rather coarse due to the convolutions in the classification networks.

Architecture selection for the global explanation stage is based on three criteria: First, the model’s attention should be mainly concentrated on the target, i.e., brain area. Therefore, we accepted at most 50% attention focused on non-biological image structures. As the networks were constructed with a single output node architecture, the memorization of a single set of features is enforced during the learning procedure. We therefore accepted diverging performances for the two groups being more lenient with the control group. The architectures Sequence 1, OhNet, Med3D, and RiekeNet fail to meet this criterion (Fig. 3) and are eliminated from further consideration. Second, the between-subject STD of the CoM should be at least an order of magnitude smaller than the image resolution (cf. Supplementary Table 1) to indicate convergence across subjects (Table 1). The three remaining architectures fulfil this criterion. Third, clearly distinguishable CoMs distributions might indicate specific features for each group, which favours Sequence 1, Med3D10, BrainID, and ResNet (Table 1, bold) but excludes Mixed Conv. Taken together, the saliency maps generated by BrainID (Fig. 4a&b) and ResNet18 (Fig. 5a&b) remain as the ones plausible enough to warrant further study of their global characteristics.

## Global explanation: Consistent saliency maps between groups within architectures

The third stage aims to identify consistent brain areas where high network saliency differs significantly between the two clinical groups. First, the two remaining architectures, BrainID (Fig. 4) and ResNet18 (Fig. 5), are considered separately. Brain areas with differing saliency in the schizophrenia group (cf. Figs. 4a and 5a) than in the control group (cf. Figs. 4b and 5b) indicate candidate regions for schizophrenia pathology (cf. Figs. 4c and 5c). For both architectures, we found one contiguous cluster surviving the correction for multiple comparisons (cf. Figs. 4d and 5d).

**Fig. 6:**
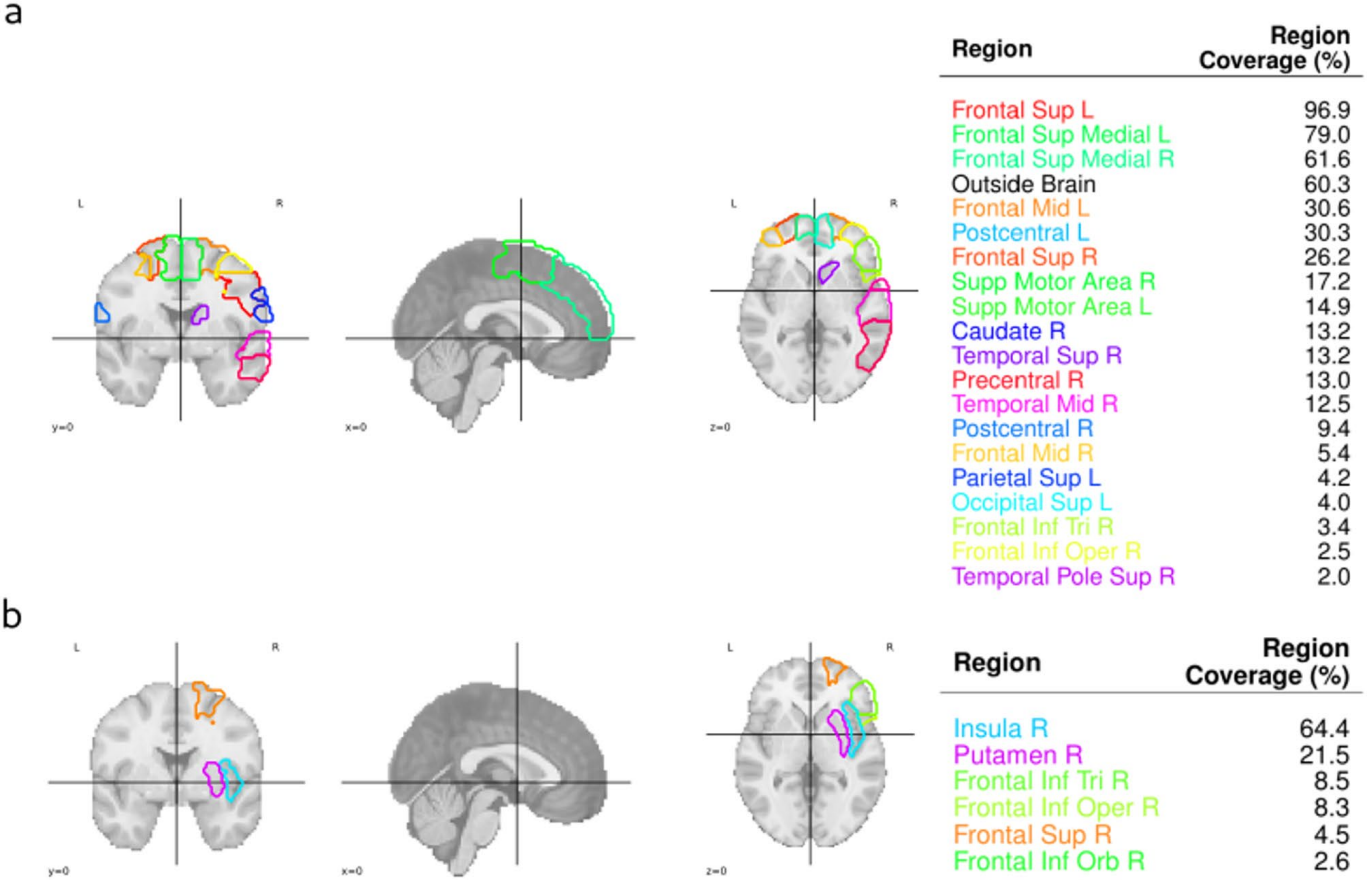
AAL atlas^30^ regions (in MNI152 space^31^ associated with the brain areas of the most robust difference between clinical groups for **(a)** BrainID and **(b)** ResNet18. For an association, at least one voxel of the top 1% voxels within the cluster derived after multiple comparison correction (Figs. 4d and 5d) must be present in the atlas region. Regions are thresholded at 2%.

Mapping the voxels with the top 1% *t*-values of these clusters to the anatomical regions of the AAL atlas^30^, reveals predominantly frontal regions with dominance of right-sided regions for both architectures (Fig. 6), even though the regions with the highest coverage for BrainID are dominated by left side regions. BrainID includes bilaterally further cortical and subcortical regions while ResNet is restricted to frontal and subcortical regions in the right hemisphere. Subcortical regions are restricted to the right side in both architectures. We find a correspondence in regions between the architectures in superior and inferior frontal regions of the right hemisphere.

## Global explanation: Intersection across architectures

**Fig. 7:**
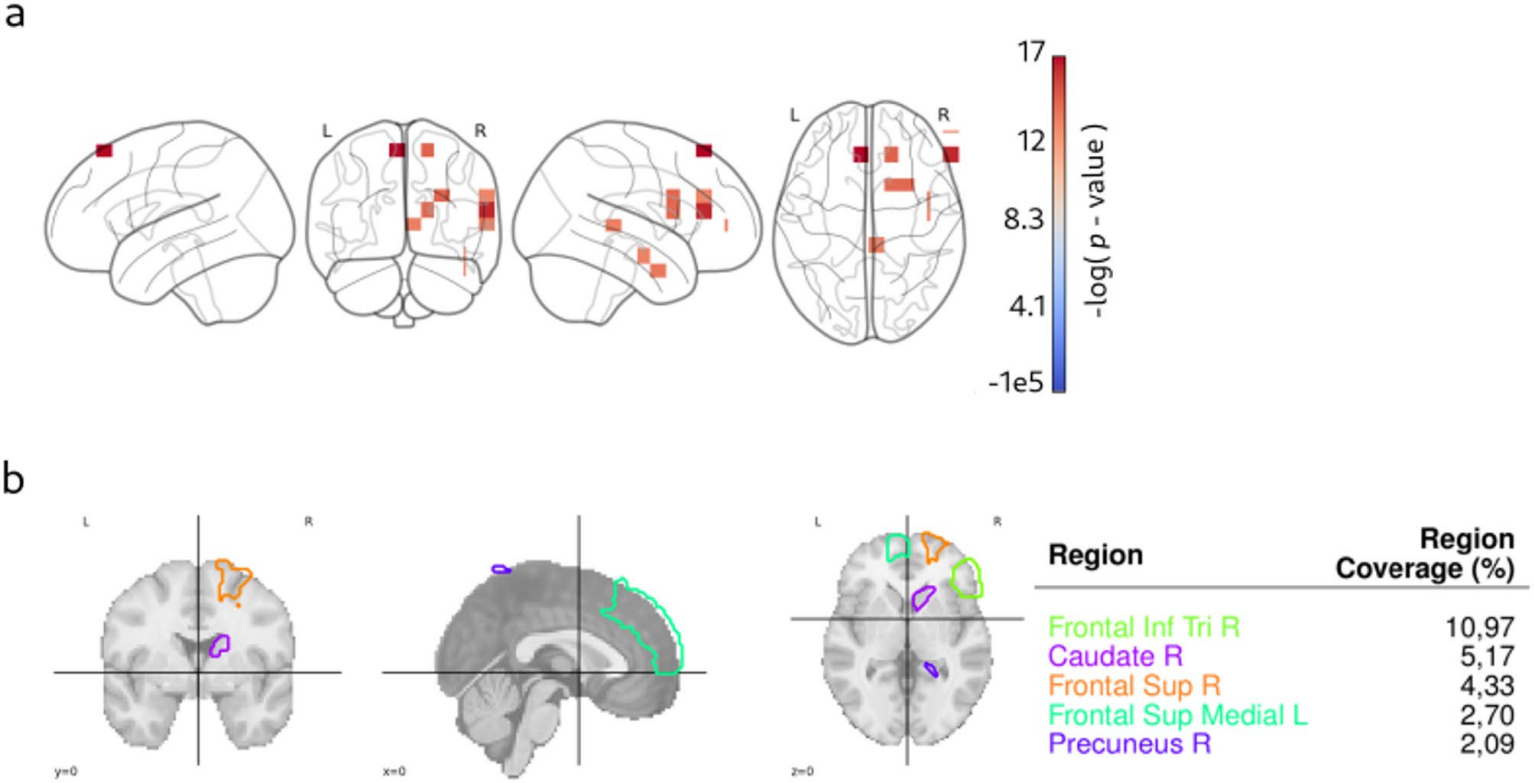
Intersection of both network types. **(a)** Overlap of areas (in MNI152 space) with higher saliency in schizophrenia than control across the architectures ResNet18 and BrainID. **(b)** AAL atlas regions associated with regions displayed in a. For an association, at least one voxel of the top 1% voxels intersecting both architectures must be present in the atlas region. Regions are thresholded at 2%.

Consensual saliency across architectures potentially indicates similar learned features, i.e. strengthen the possibility of finding a disorder-relevant brain area. Even though some of the anatomical regions associated with the top 1% of voxels within each architecture overlap (Fig. 6.), we do not find any top 1% voxels overlapping across the two architectures. Therefore, we relax our threshold for the intersection analysis and consider all voxels in the one contiguous cluster of each classifier (Figs. 4d and 5d). From this overlap, we now consider only the top 1% voxel again (Fig. 7a). Mapping those voxels to the AAL atlas reveals predominantly frontal regions again and a dominance of the right hemisphere (Fig. 7b). The two regions that are mapped for all three variants, the two individual architectures and the intersection, are the right superior frontal gyrus and the triangular part of the right inferior frontal gyrus.

## Discussion

In this three-stage approach, we demonstrate the necessity and feasibility of transparency in the decision process of DL-architectures for image-based decision support in psychiatry. Furthermore, we demonstrate the value of a local explainability method, which is a helpful tool for enriching individual decisions made by a DL-based clinical decision support system, in deriving global anatomical biomarkers for a psychiatric disorder such as schizophrenia.

Seven models, all based on DL-architectures frequently used for medical image analysis and adapted for the analysis of 3D MRI images, achieve a good classification performance with areas under the curve ranging from 0.75 to 0.85. The achieved accuracies correspond well to other ML-models based on anatomical MRI for the task of schizophrenia diagnosis^8^. However, the evaluation of their saliency maps obtained with Grad-CAM provides rather diverse performances with respect to the plausibility of the models, with some models even basing their decision primarily on areas outside of the brain.

Saliency maps are tools most often used for qualitative evaluation and visualisation of the decision process of classification models whereas their quantitative evaluation is often considered unsuitable and is scarcely conducted^32,33^. In image classification tasks in which the face validity of the produced saliency map can be easily assessed visually or *via* a well defined ground truth area of interest, a quantitative evaluation might not be necessary or hard. The anatomical changes in schizophrenia are subtle, distributed^34–38^, and still an ongoing matter of research. Ground truth is therefore not possible. The quantitative metrics developed in this study are based on the rather coarse “region of interest” that includes the whole brain area, the assumption that the saliency should not be uniformly distributed, and that similar brain regions are affected in the majority of patients. Our approach can be generalized to other disorders characterized by subtle, complex brain alterations as it is typical for many psychiatric disorders. For biomarker discovery, further analyses that detect confined clusters of intensity can be a valuable addition to the analyses presented in this study.

The anatomical regions most frequently identified in reviews or meta-analyses of neuroimaging studies on schizophrenia patients^34–38^ are frontal^39^, temporal^40,41^, and subcortical^42^ regions. For the data set used in this study, frontal, temporal, and insular grey matter reduction was assessed with voxel based morphometry^28^. Along this line, frontal regions are highlighted most prominently by the saliency maps of both our classifiers as well as their intersection. Beyond the right superior frontal gyrus and the triangular part of the right inferior frontal gyrus, however, the specific regions vary across classifiers. While the saliency map of BrainID highlights left superior and right temporal regions as well, the map of ResNet18 emphasizes the right insula and putamen. The insula has been discussed for its role in the progression of schizophrenia^43,44^ and has also been highlighted in another study utilizing saliency maps^20^. The basal ganglia and related subcortical structures are affected in several psychiatric disorders with caudate nucleus and putamen, the pertaining regions highlighted in our results, being specifically involved in schizophrenia^42^. Indeed, the right caudate has been found to be enlarged in schizophrenia patients^34,42^. The correspondence of the saliency maps from this study to findings in the current body of literature confirms the plausibility of our results and supports the approach introduced.

The saliency maps generated for this study lack precision and regional focus. This becomes especially apparent on the architecture types that were not further analyzed. One reason for this problem might be the insufficient performance and generalization of the classifiers. The small sample size can be one contributing factor to the inadequate generalization ability. However, even a model performance does not guarantee that a model has captured a genuine underlying relationship^45^. Consistent with these findings, our CNN experiments achieve very similar accuracy scores despite producing highly divergent saliency maps for most models. Hence, implausible saliency maps might indicate a dataset memorization.

Based on the learnings from the current study, upcoming work might strive for a region-focused saliency map generation by improving the classification performance, construct networks additionally capturing regions of interests e.g. segmentation networks or including saliency map related metrics in the training cost function. Due to the method-inherent smoothing of the Grad-CAM method, the conducted brain region mapping procedure might also be imprecise and thereby lead to slight distortions in region coverage.

To conclude, the generalizable approach employed in this work is a first step to enable the identification of regions of high relevance during the classification of pathologies by transitioning from local saliency explanations to accumulated global information. The anatomical findings of this study converge with findings of classical imaging studies on schizophrenia patients, giving the approach plausibility.

### Methods

#### Data

The data used in this study was obtained from the MCIC collection^28^ in July 2019. The collection contains structural T1-weighted MR images of 158 adult SC patients and 169 demographic, age, and sex-matched HC. Four research sites were involved in the data collection process from 2004 to 2006. All subjects provided informed consent to participate in the study that was approved by the human research committees at each of the sites. Patients had to be diagnosed with SCZ conforming to the Diagnostic and Statistical Manual of Mental Disorders, 4th Edition (DSM-IV)^46^. We included only data from sites A, C, and D because the images originating from site B were not publicly released due to IRB restrictions. Furthermore, the data from ten subjects failed transformation to BIDS format^47^ due to missing meta-data, leaving a subset of 101 SCZ and 91 HC for this study (Table 2).

**Table 2:** Sample characteristics of the MRI data set per class (Schizophrenia, Control).

	Schizophrenia	Control
Number of subjects	101	91
Age (years) mean ± STD	34.2 ± 11.2	32.3 ± 11.8
Gender (male/female)	78/23	61/30

The brain images were skull-stripped and registered to MNI 1 mm^3^isovoxel with the nypipe toolbox developed by Gorgolewski et al.^48^. Image intensities in the 1% and 99% percentile were removed; empty image slices deleted and the image intensity distribution was normalized to −1 to 1. Since training time and complexity rises with increasing image size, the image size was down-scaled to 2 mm^3^ isotopic resolution resulting in 643 voxels per image. If pretrained network weights were used, the corresponding network requirements were recreated in order to mimic the original training circumstances as far as possible and to increase transferability of the pretrained network weights (Supplementary Table 1).

## Deep learning architectures

During this study, seven architecture types with and without pretrained weights were attuned to distinguish schizophrenia patients from a control group. Sequence 1, a three convolutional blocks deep 3D CNN, inspired by the VGG-16 architecture^22^, was designed and trained from scratch (Supplementary Images 1). The second fully trained network, OhNet, is a reimplementation of one of the best performing 3D DL architectures in the field of schizophrenia classification^23^ with an adapted single output layer. In all pretrained architectures, the final network layer was replaced with two additional dense layers for information processing. The networks Med3D^24^, a pretrained ResNet-10 3D adaptation for medical image analysis, and RiekeNet^26^, a four layers deep network for Alzheimer’s detection, were fine tuned without any further adaptations. In the case of BrainID fine tuning, the U-Net encoder of the network was used, as suggested by the authors for any downstream task requiring brain feature extraction. For the usage of the video processing networks MixedConv^27^ and ResNet^27^, the first convolutional layer had to be replaced. As a suitable initialization, unadjusted weights were set to the average of the original convolutional weights. All architectures result in a single output node.

### Deep learning classifier training

During the training process, the network performance was enhanced as much as possible while having the most robust training course. Every architecture type was trained with an AdamW optimizer using a batch size of nine images. If needed, default weight decays were optimized to achieve a smoother loss progression. When applicable, learning rate and epoch number were extracted from the original publications and subsequently adapted manually. Every CNN architecture was trained for at least 20 epochs until the convergence of the validation loss. When pretrained weights were available, transfer learning was applied. Fine-tuning of pretrained network layers did not prove beneficial and hence was not conducted. As a network regularization, dropout within the last fully connected layers was used. The dropout ratio was increased until a decrease in the validation accuracy was registered. A detailed record of all hyper parameters can be found in Supplementary Table 2 of the supplemental material. Validation accuracies were measured using a stratified five-fold cross validation using a 80%/20% train/validation split. For each architecture, the model trained on the best performing fold was selected for the subsequent local and global analysis stages.

### Local explanation: saliency map generation

The saliency maps generated in this work were obtained by extending the original 2D Grad-CAM method proposed by Selvaraju et al.^19^ to our 3D MRI images. The generated saliency maps are class selective and applicable without further architecture adaption, and therefore suitable for comparing and aggregating saliency information across a variety of CNN-architectures. This is a prerequisite for subsequent model selection as well as for extracting the required global explanations. Since all used networks examined in this study are constructed with a single output node, the control class is captured as a so-called counterfactual explanation. For this study, no normalization or intensity scaling of the generated saliency map values was applied. The resulting saliency map resolutions were rescaled to the input image size using nearest neighbor interpolation. The saliency maps used for the further analysis were generated from the validation set of the best-performing fold for each respective CNN-architecture. Since saliency maps based on incorrect image classifications might highlight regions that do not support a correct prediction, images with incorrect classification were generally excluded from the analysis.

### Local explanation: saliency map evaluation

To assess the plausibility of individual decisions several metrics were obtained. In a first visual assessment individual saliency maps were averaged per CNN-architecture and per class. A plausible saliency map should be concentrated on the brain area of the image. Ideally, the attention should be focused on confined areas to indicate the differences in the localization of attention between the schizophrenia and the control group.

Next to the visual examination of the generated saliency maps, quantitative metrics such as the mass accuracy and the center of mass were applied. The MA ascertains that plausible classification predictions are based on voxels located within the brain area. A high concentration of attention in other regions, e.g., the image borders, would suggest the presence of a non-identified bias. For the calculation of the MA, the saliency map is compared with a ground truth^18^. The metric depicts the amount of attention within the area of the ground truth in contrast to the sum of attention outside the region of interest. A conservative mask around the brain area including padding was chosen as a ground truth to account for attention blurring caused by the filter and pooling sizes of the last convolutional layer targeted by the Grad-CAM.

As a second evaluation metric, the CoM of each saliency map was calculated in order to assess the differences between schizophrenia and control groups. When looking at a convex-shaped area of attention or a multicentered attention map the CoM cannot capture the true nature of the distribution. Within a homogeneously spread attention map the CoM would be concentrated in the image center. For this reason the calculated CoM can not be interpreted as positions with high network attention. Based on the aforementioned metrics, two CNN architectures were selected for further evaluation.

### Global explanations: regions of stable network attention

Though saliency maps provide a good local explanation for individual decisions, they do not provide any insight into systematic, recurring features of the disease. Based on the assumption that structural brain alterations in schizophrenia would influence the classifiers decision and thereby cause stable attention patterns in the networks saliency maps, we conducted a voxel-wise, two-sided t-test. The testing strategy thereby captures the global differences of saliency between schizophrenia patient images and control subjects.

In order to define areas of high consistency and reliability the multi-comparison problem was tackled with a TFCE-Error correction^29^ using 20,000 iterations. The resulting p-map was additionally bonferroni corrected with an alpha of 0.0001 and reduced to the biggest connected cluster of voxels to ensure significance. Significant voxels are matched to locally corresponding AAL atlas regions^30^. Per region the number of hits is counted. During hit counting, no minimal number of hits per region was set. As it is inherently part of a convolution, the information gained through the Grad-CAM method is not precisely localized. Consequently, not only the region associated with the actual voxel position was considered, but also matches of neighboring voxels were counted proportionately. This second hit is evenly distributed over all voxel-adjacent regions. For a more region-size sensitive interpretation, the hit coverage per region was calculated.

In the search of reliable biomarkers, consensual regions with high and stable network attention would suggest a higher probability of a true underlying correlation within the data. Therefore the most stable regions of high network attention after two-staged error correction were intersected and mapped to associated AAL atlas brain regions.

## Supplementary Information

Below is the link to the electronic supplementary material.

Supplementary Material 1

## Data Availability

The MRI data used in this study is available from the MCIC collection upon request via COINS data sharing website (https://www.nitrc.org/projects/coins/). All pretrained network weights are provided as open-source models by the authors of the cited publications.
