# Supplementary material for: Decision processes in 3D structural MRI schizophrenia classification evaluated with saliency maps
Source: Sci Rep. 2026 Jun 13;16:18362. doi: 10.1038/s41598-026-57667-z (PMC13264623; doi:10.1038/s41598-026-57667-z)
Supplement: Supplementary file 1 — Supplementary Material 1 [file 41598_2026_57667_MOESM1_ESM.pdf]

Supplementary Material to

Decision processes in 3D structural MRI schizophrenia classification evaluated with saliency maps

Julia Jelitzki [12], Alexandra Reichenbach [12*+], and Alexander Windberger [3+]

[1]Center for Machine Learning, Heilbronn University, Heilbronn, Germany
[2]Medical Faculty Heidelberg, University of Heidelberg, Heidelberg, Germany
[3]Faculty of Informatics, Heilbronn University, Heilbronn, Germany
[*] corresponding author: alexandra.reichenbach@hs-heilbronn.de
[+] these authors contributed equally to this work

Keywords: deep learning, schizophrenia, structural magnetic resonance imaging, explainable AI, Grad-CAM, saliency map

Images

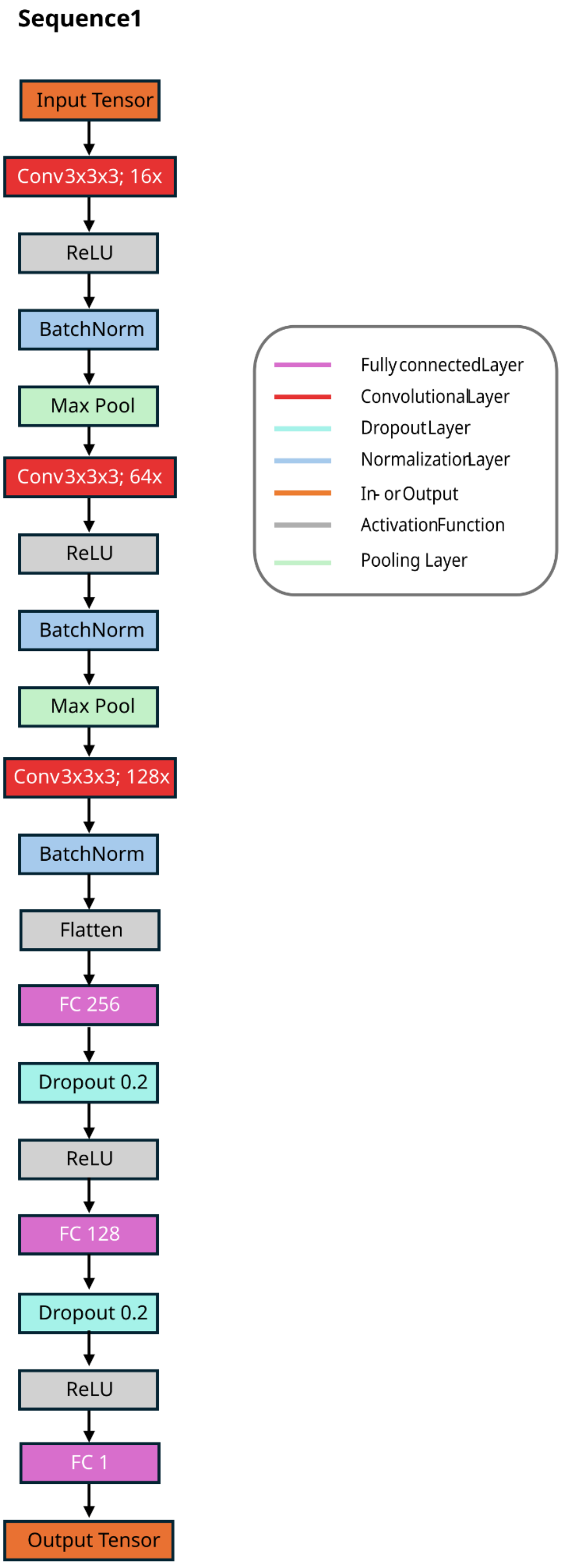

Supplemental Figure 1: Architecture depiction of Sequence 1 architecture.

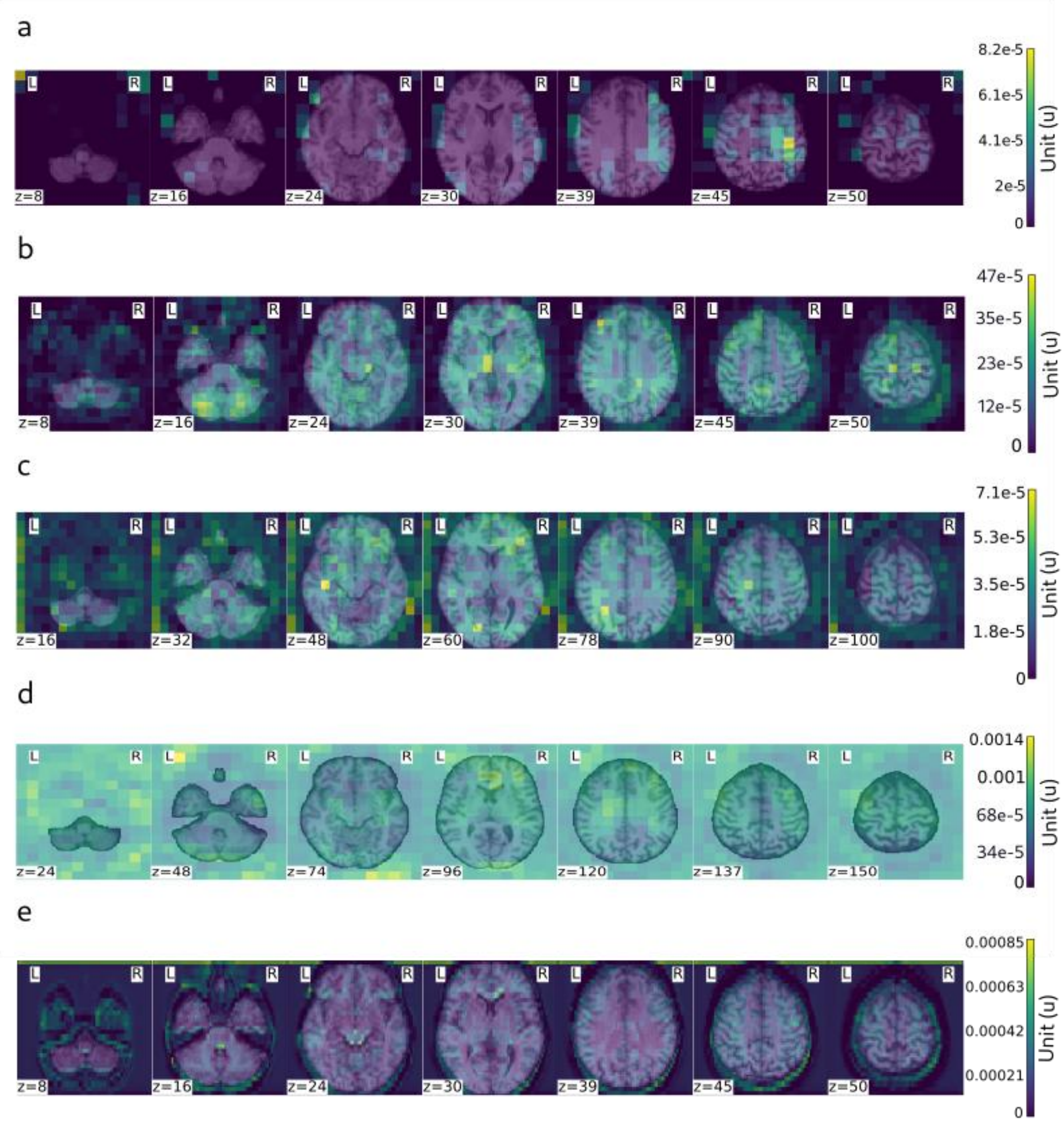

Supplemental Figure 2: Averaged saliency map of control class for the architecture types a) Sequence 1 b) OhNet c) Med3D d) RiekeNet e) MixedConv. Note that the architecture styles require different image resolutions.

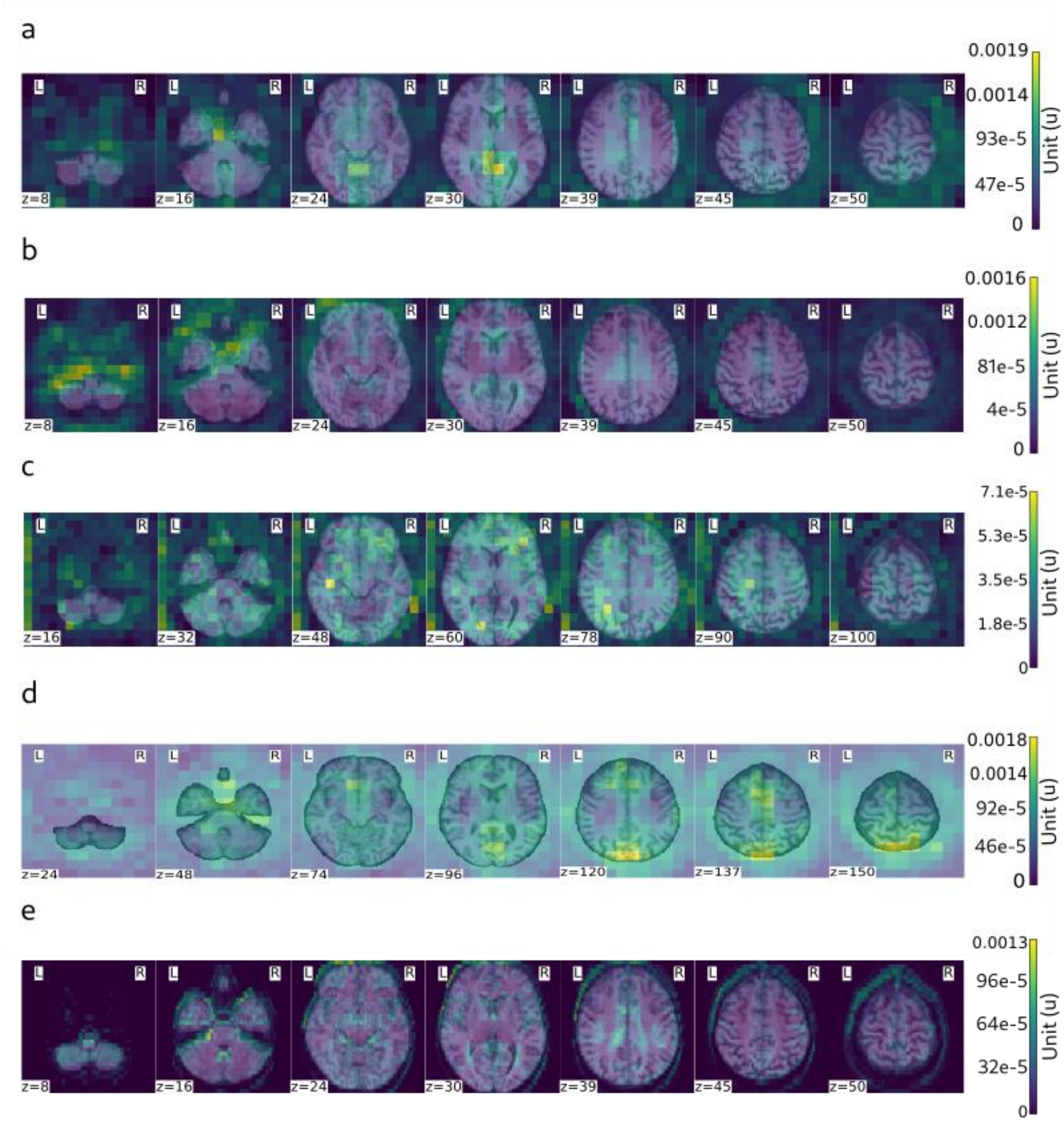

Supplemental Figure 3: Averaged saliency map of patient class for the architecture types a) Sequence 1 b) OhNet c) Med3D d) RiekeNet e) MixedConv. Note that the architecture styles require different image resolutions.

Tables

Network Name	Input Size	Architecture Type	Preprocessing	Pretrained Weights	Original Training Data
Sequence 1	64x64x64	Sequencial 3D	None	no	None
OhNet	64x64x64	Sequencial 3D	None	no	None
Med3D10	128x128x128	ResNet 3D	*z*-normalisation	yes	Medical
BrainID	128x128x128	U-Net 3D	rescale 0- 1	yes	Medical
RiekeNet	192x220x192	ResNet 3D	*z*-normalisation	yes	Medical
Mixed Conv	64x64x64	Mixed Convolution 3D	rescale 0-1 *z*-normalisation	yes	Generic
ResNet18	64x64x64	ResNet 3D	rescale 0-1 *z*-normalisation	yes	Generic

Supplementary Table 1. Training details and additional image pre-processing procedures for every network architecture. All images were additionally prepared (skullstripped, MNI space registration, ect.) as declared in the Data section.

Network Name	Lerningrate	Dropout	Weight Decay	Epochs	Pretrained Weights	Trainable Parameters
Sequence 1	0.00015	0.2	0.00001	25	no	348.705
OhNet	0.00007	0.2	0.01	20	no	746.544
Med3D10	0.000005	0.35	0.01	20	yes	536.877.185
BrainID	0.00002	0.6	0.05	30	yes	262.366.185
RiekeNet	0.0009	0.15	0.01	20	yes	18.432.003
Mixed Conv	0.009	0.3	0.5	30	yes	150.657
ResNet18	0.00094	0.3	0.05	30	yes	150.657

Supplementary Table 2. Overview of key network training parameters for all architecture types.
